# Live Birth Rate Following Bed Rest *Versus* Early Mobilization After Embryo Transfer: A Systematic Review And Meta-Analysis

**DOI:** 10.5935/1518-0557.20220003

**Published:** 2022

**Authors:** Jorge Rodriguez-Purata, Maitane Alonso-de Mendieta, Maria Jose Gomez-Cuesta, Enrique Cervantes-Bravo

**Affiliations:** 1 Clinica de la Fertilidad “CdelaF”, Colonia Santa Fe, Cuajimalpa de Morelos, Mexico City, Mexico; 2 Centro Medico ABC, Colonia Santa Fe, Cuajimalpa de Morelos, Mexico City, Mexico

**Keywords:** live birth rate, embryo transfer, *in vitro* fertilization, early mobilization, post-transfer bed rest

## Abstract

Embryo transfer (ET) is the final step of in vitro fertilization (IVF). Different strategies have been proposed to increase the likelihood of implantation, such as post-transfer bed rest. The objective of this manuscript was to compare the clinical outcomes of embryo transfers after IVF of patients offered rest vs. early ambulation. The patient, intervention, comparison, and outcome(s) (PICO) model was used to select the study population, which included women/couples submitted to IVF and prescribed bed rest or early ambulation. Only studies including live birth (LB) as an outcome were included (www.crd.york.ac.uk/PROSPERO/CRD42020188716) A systematic search for studies was conducted on MEDLINE, ClinicalTrials.gov, PubMed, and the Cochrane Library. A librarian coordinated the searches in May 2020, which considered articles published since 1995. All original peer-reviewed articles in English were included, regardless of study design. The search retrieved 27 citations, of which 14 were eligible for full-text analysis and four accepted for inclusion. The studies included data on 21,598 patients/cycles (rest: 20,138; early ambulation: 1,460). Patients prescribed bed rest had an LB rate of 43.6% *vs*. 52.5% in the individuals not offered bed rest. The meta-analysis yielded an odds ratio of 0.77 (95% CI 0.5-1.2), which means patients on bed rest were 23% less likely to have a LB; nevertheless, this difference was not statistically significant. Considering that there is no difference between the two strategies, there is no evidence to recommend bed rest after embryo transfer.

## INTRODUCTION

Reproductive medicine has radically evolved since the first in vitro fertilization (IVF) cycle was reported in 1978 (Steptoe & Edwards, 1978), mainly due to the great advances observed both at the clinical (Macklon *et al.*, 2006) and laboratory levels (Niederberger *et al.*, 2018). As a result, more emphasis has been placed on the optimization and standardization of the embryo transfer (ET) procedure through the development of evidence-based protocols. Although debated, one of the strategies adopted to increase the success rate of IVF cycles is post-transfer bed rest (Sallam, 2005; Abou-Setta *et al.*, 2014).

ET comprises placing the obtained embryos in the uterus of a patient. This is the final, and in some respects the most critical step of a sequence of events that transpire during an IVF cycle. Post-transfer uterine contractions that might potentially affect embryo implantation at the deposition site have been reported (Fanchin *et al.*, 1998; Lesny *et al.*, 1998). Expulsion of the transfer fluid has been observed after ET (Schulman, 1986; Ghazzawi *et al.*, 1999), while other authors have reported that the transfer content is not affected by patient ambulation immediately after transfer (Lambers *et al.*, 2009). For this reason, various interventions have been proposed, such as post-transfer rest (Orvieto *et al.*, 1998), to potentially resolve this situation.

In this regard, several studies, both prospective and retrospective, have evaluated this intervention with contrasting results, with a greater tendency towards no evidence of benefit in post-transfer bed rest (Abou-Setta *et al.*, 2014). Despite these results, patients and their treating physicians continue to recommend limiting post-transfer physical activity (Hawkins *et al.*, 2014) with no real evidence of improvement in clinical outcomes. Other studies have reported negative effects of bed rest (Küçük, 2013). Importantly, no previous study or meta-analysis has included the live birth rate (LBR) as a primary outcome (Craciunas & Tsampras, 2016; Cozzolino *et al.*, 2019).

Given the above, we conducted a systematic review of the literature and a meta-analysis of the results of studies to determine whether post-transfer rest had an impact on the LBR of patients submitted to IVF cycles.

## MATERIAL AND METHODS

### Protocol and registration

**Figure 1:**
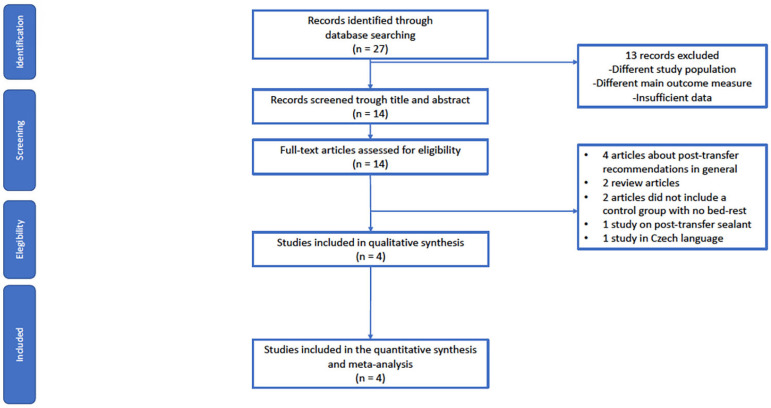
PRISMA*,** flow diagram. * PRISMA: Preferred Reporting Items for Systematic Reviews and Meta-Analyses ** Moher et al., 2009a;b

The study adhered to the principles of the preferred reporting guidelines for systematic reviews and meta-analyzes (PRISMA) (Moher *et al.*, 2009a;b) (Figure 1). The study protocol was registered at http://www.crd.york.ac.uk/PROSPERO/CRD42020188716 before it was started. This study did not require institutional review board approval, since it is a systematic review.

### Eligibility criteria

The patient, intervention, comparison and outcome(s) (PICO) model was used to select the study population (Schardt *et al.*, 2007). Women/couples who underwent IVF cycles were included, and the outcomes of patients prescribed bed rest *vs*. early ambulation were compared.

### Search strategy

The authors systematically searched the literature for studies that matched the clinical question on MEDLINE, ClinicalTrials.gov, PubMed, and the Cochrane Library. A combination of Medical Subject Heading (MeSH) terms and/or relevant text words was performed to compare the clinical outcomes of rest *vs*. no post-transfer rest in IVF. The authors also checked citations on the Web of Science and manually searched the references of the articles. The searches were coordinated in May 2020. Updates to the search were made in June 2020. All records were evaluated for eligibility by two independent reviewers (J.R-P./E.C-B). All original peer-reviewed articles were included, regardless of study design.

The searches in electronic databases included the following combined search terms: (“Fertilization in Vitro” OR “in vitro fertilization” OR IVF OR “Reproductive Techniques, Assisted” OR “Oocyte Retrieval” OR “Egg Collection”) AND (“Bed Rest” OR “rest” OR “Bed Rest/adverse effects*” OR “Bed Rest/statistics & numerical data” OR “Postoperative period” OR “Early Ambulation) AND (“Embryo Transfer” OR “Embryo Transfer, Methods” OR “Embryo Implantation”) AND (“Live Birth Rate” OR “Live Birth” OR “Pregnancy Outcome” OR “Pregnancy” OR “Treatment Outcome” OR “Infant, Newborn” OR “Birth Rate” OR “Live-Birth”).

### Study selection

Two authors (J.R-P./E.C-B) independently assessed article titles and abstracts. Duplicates were removed using the Zotero software and manually. The final decision to include/exclude articles was made after they were examined in their entirety. Discrepancies were resolved by discussion and consensus among the authors, with the participation of another author (MJ.G-C). To acquire the highest level of evidence, the authors selected randomized trials and observational studies, including cohort and case-control studies. Trials published only as abstracts, letters to the editor, editorials, or studies withdrawn from the literature after publication were excluded. Systematic reviews were also excluded, but references were checked first.

Intervention studies were eligible if: 1) they were randomized controlled trials (RCT), not RCT, prospective, retrospective observational studies, or cohort studies; 2) evaluated rest vs. no rest; 3) reported the LBR or clinical pregnancy rates (CPR) as outcome measures.

### Data extraction and quality assessment

If the records were eligible, two reviewers (J.R-P. And E.C-B) collected and imported the data into an electronic database. The following data were captured: year of publication, study design, study period, intervention, number of patients (total, intervention, and comparator), age, rest time, biochemical pregnancy rate (BPR), CPR, LBR, and miscarriage rate (MR). Furthermore, for intervention studies, allocation concealment and blinding were also recorded.

The primary endpoint of the present meta-analysis was LBR. Although it could be argued that the implantation rate might be a better indicator of the effect of bed rest due to its temporality, the authors considered that the optimal clinical objective of an IVF cycle is the birth of a healthy infant that is ultimately sent home with their parents. Furthermore, the authors considered that the decision to prescribe rest or early ambulation might potentially impact the probability of miscarriage. As secondary endpoints, CPR and MR were studied. A LB was defined as any event of LB of a live product of conception after 24 weeks of gestation. LBR was defined as the proportion of LBs in relation to included patients. A clinical pregnancy was defined as the visualization of a gestational sac with a heartbeat after embryo transfer. CPR is the proportion of clinical pregnancies in relation to included patients. A biochemical pregnancy was defined as a blood pregnancy test >5mIUI/mL. The BPR is the proportion of biochemical pregnancies in relation to included patients. A miscarriage was defined as the loss of pregnancy before the 20^th^ week of gestation. MR was defined as the proportion of abortions in relation to the number of confirmed pregnancies.

### Appraisal of certainty of evidence

Two authors (J.R-P./E.C-B.) Independently assessed the risk of bias of each study and the methodological quality of the included studies using the criteria described in the Cochrane Handbook for Systematic Reviews of Interventions (Higgins & Thompson, 2002). In addition, seven specific domains related to risk of bias were assessed: random sequence generation, allocation concealment, blinding of participants and staff, blinding of outcome assessment, incomplete results, selective data reporting, and other biases. Authors’ judgments were expressed as “low”, “high” or “unclear” risk of bias. The evaluation was carried out independently by two reviewers (J.R-P., E.C-B) and disagreement were resolved by discussion between the two parties including a third reviewer (MJ.G-C.).

### Statistical analysis

Clinical outcomes were collected as dichotomous data. The results of the studies were combined in the meta-analysis using a Mantel-Haenszel fixed effect model for a pooled odds ratio (OR) and a 95% confidence interval (CI). Forest plots and the I^2^ statistic were calculated for each study outcome and each group as a way to quantify the statistical heterogeneity of the included studies (Eden *et al.*, 2011). I^2^ was defined as: 0% to 40%: may not be important; 30% to 60%: may represent moderate heterogeneity; 50% to 90%: may represent substantial heterogeneity; 75% to 100%: considerable heterogeneity (Higgins & Thompson, 2002; Higgins *et al.*, 2011). All calculations were performed with RevMan 5.4 (Review Manager, version 5.4, The Cochrane Collaboration, 2020).

## RESULTS

### Study selection

The search retrieved 27 citations, of which 14 were deemed eligible for review and four accepted for inclusion (Sharif *et al.*, 1998; Bar-Hava *et al.*, 2005; Purcell *et al.*, 2007; Gaikwad *et al.*, 2013) (Figure 1). Data from a total of 21,598 patients/cycles (rest: 20,138; early ambulation: 1,460) were included.

### Description of included studies

Of the included studies, one was from the UK (Sharif *et al.*, 1998), one from Israel (Bar-Hava *et al.*, 2005), one from the USA (Purcell *et al.*, 2007), and one from Spain (Gaikwad *et al.*, 2013). There were two randomized controlled trials (Purcell *et al.*, 2007; Gaikwad *et al.*, 2013), one prospective (Bar-Hava *et al.*, 2005), and one retrospective study (Sharif *et al.*, 1998). Table 1 shows the description of the studies.

**Table 1:** Description of included studies.

	Design	Randomization	Luteal Phase Support	ET day	ET catheter	Bed-Rest / no Bed-Rest			
						(minutes)	Patients (n)	Age (years)	Number of Embryos Transferred
Sharif *et al.*, 1998	Retrospective	No	100mg/day IM	2, 3	Embryon®	NA / 0	19697/ 1091	NA	NA
Bar-Hava *et al.*, 2005	Prospective	No	NA	3	NA	60 / 0	239/167	34.2 / 34.2	2.9 / 2.7
Purcell *et al.*, 2007	RCT	Yes Numbered opaque envelope	IM (dose NA)	2, 3, 5	Wallace®	30 / 0	82/82	36.9 / 36.8	3.3 / 3
Gaikwad *et al.*, 2013	RCT	Yes Computer	800mg/day PO	3, 5, 6	Wallace®	10 / 0	120/ 20	41.2/ 40.9**	2 / 2

ET: embryo transfer; n: sample size; RCT: randomized controlled study; NA: no information; IM: intramuscular; PO: orally

### Assessment of the risk of study bias

**Figure 2:**
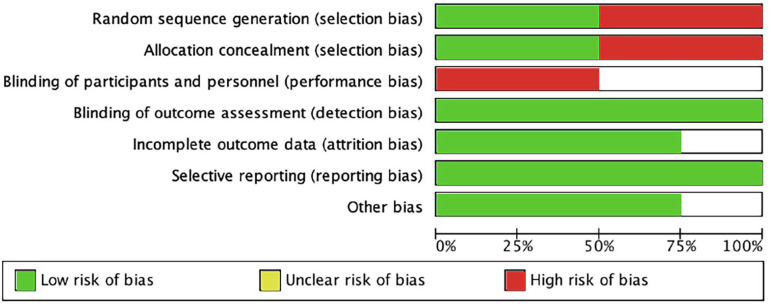
Risk of bias graph: authors' judgments about each element of risk of bias, presented as percentages.

According to the guidelines suggested by the Cochrane Collaboration, the quality of the included studies was moderate (Figure 2).

### Live birth rate

**Figure 3:**
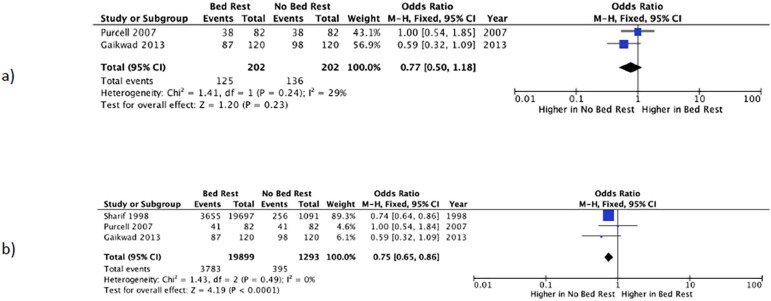
Forest plot of the comparison of bed rest vs. no bed rest after embryo transfer. Analysis: a) Live Birth Rate; b) Clinical Pregnancy Rate.

Two studies reported LBR (Purcell *et al.*, 2007; Gaikwad *et al.*, 2013). Patients prescribed bed rest after ET had an LBR of 43.6% *vs*. 52.5% in patients prescribed early ambulation (Table 2). The meta-analysis of the two studies yielded an OR 0.77 ((95% CI 0.5-1.2), I^2^=29%), which implies a 23% lower chance of having a LB among patients prescribed bed rest, although the difference was not statistically significant (Figure 3).

**Table 2:** Clinical outcomes by study group and total.

	Live Birth Rate		Clinical Pregnancy Rate		Biochemical Pregnancy Rate		Miscarriage Rate	
	Bed Rest	No Bed Rest	Bed Rest	No Bed Rest	Bed Rest	No Bed Rest	Bed Rest	No Bed Rest
Sharif et al., 1998	-	-	18.6% (3655/19697)	23.5% (256/1091)	18.9% (3722/19697)	29.5% (322/1091)	-	-
Bar-Hava et al., 2005	-	-	-	-	21.3% (51/239)	24.6% (41/167)	-	-
Purcell et al., 2007	46.3% (38/82)	46.3% (38/82)	50.0% (41/82)	50.0% (41/82)	-	-	7.3% (3/41)	7.3% (3/41)
Gaikwad et al., 2013	41.7% (50/120)	56.7% (68/120)	-	-	69.2% (83/120)	75% (90/120)	39.8% (33/83)	24.4% (22/90)
All	43.6% (88/202)	52.5% (106/202)	18.7% (3696/19779)	25.3% (297/1173)	19.2% (3856/20056)	32.9% (453/1378)	27.8% (36/124)	19.1% (25/131)

### Clinical pregnancy rate

Three studies reported CPR (Sharif *et al.*, 1998; Amarin & Obeidat, 2004; Purcell *et al.*, 2007). Patients prescribed bed rest had a CPR of 18.7% *vs*. 25.3% in subjects prescribed early ambulation (Table 2). The meta-analysis of these three studies yielded an OR of 0.75 ((95% CI 0.7-0.9), I^2^=0%), which implies a 25% lower chance of clinical pregnancy among patients prescribed post-transfer rest (*p*<0.0001) (Figure 3).

### Biochemical pregnancy rate

**Figure 4:**
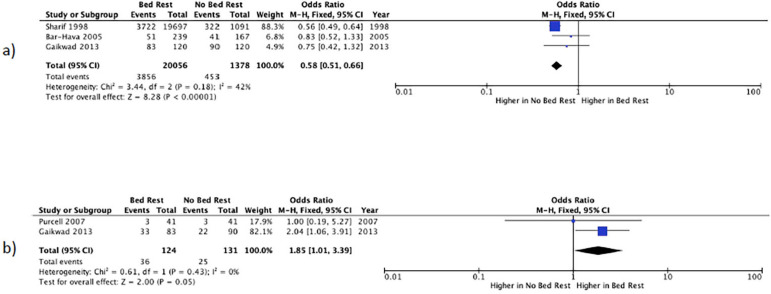
Forest plot of the comparison of bed rest vs. no bed rest after embryo transfer. Analysis: a) Biochemical Pregnancy Rate; b) Miscarriage Rate.

Three studies reported BPR (Sharif *et al.*, 1998; Bar-Hava *et al.*, 2005; Gaikwad *et al.*, 2013). A BPR of 19.2% was observed in patients prescribed bed rest *vs*. 32.9% in individuals not prescribed bed rest (Table 2). The meta-analysis of these studies yielded an OR of 0.58 ((95% CI 0.5-0.7), I^2^=42%), which implies a 42% lower probability of biochemical pregnancy among patients prescribed bed rest (*p*<0.0001) (Figure 4).

### Miscarriage rate

Two studies reported MR (Purcell *et al.*, 2007; Gaikwad *et al.*, 2013), which, in patients prescribed bed rest, was 27.8% *vs*. 19.1% in subjects prescribed early ambulation (Table 2). The meta-analysis of these studies yielded an OR of 1.9 ((95% CI 1.0-3.4), I^2^=0%), which implies patients prescribed post-transfer rest are 1.9 times more likely to have an abortion (*p*<0.0001) (Figure 4).

## DISCUSSION

The present systematic review and meta-analysis is the first published in the literature that included LB as the primary endpoint. By conducting this study to assess the strength of the evidence published to date, the probability of achieving a LB was similar whether post-transfer bed rest was prescribed or not. Interestingly, the evidence showed that patients placed on bed rest immediately after transfer had a higher probability of achieving pregnancy. Nevertheless, patients prescribed bed rest also had a higher probability of having a miscarriage, ultimately yielding similar LB odds.

Since the beginning of our specialty, it has been thought that it is essential to rest after the last phase of an IVF cycle, generally without evidence. This was most likely due to the inefficiency associated with early assisted reproductive technologies. Advances in ovarian stimulation (Macklon *et al.*, 2006) and assisted reproduction laboratories (Niederberger *et al.*, 2018) eventually revealed that the vast majority of the failures to achieve pregnancy were due to deficiencies in other steps of IVF (Niederberger *et al.*, 2018), not necessarily associated with post-transfer bed rest.

Previous studies have tried to evaluate and meta-analyze the dilemma of whether performing post-transfer bed rest benefits patients. In 2014, the Cochrane Library published possible interventions that might help achieve higher success rates after an ET in IVF (Abou-Setta *et al.*, 2014), and concluded that there was not enough information to recommend bed rest. Craciunas & Tsampras (2016) performed a meta-analysis of bed rest versus no bed rest. However, in that particular study, the authors did not include LBR as the primary objective of the analysis. Additionally, the authors included two studies in which the control group underwent less bed rest instead of no bed rest. The authors concluded that bed rest was not associated with a higher CPR and that it possibly reduced the implantation rate (Craciunas & Tsampras, 2016). Finally, Cozzolino *et al*. (2019) also performed a meta-analysis including these studies themselves. Neither of the meta-analyses (Craciunas & Tsampras, 2016 and Cozzolino *et al.,* 2019) included the study carried out by Gaikwad *et al*. (2013), one of the best designed studies to include the LBR as a primary endpoint.

On the other hand, the most complicated element in changing toward a recommendation of not resting is not the generation of scientific evidence, but the patient education effort that would be required. We often see in daily practice patients asking - and sometimes demanding - that they should rest for 30 minutes or even an hour. We believe that if the results of the present study were explained to a patient, she might understand that resting does not help and might even harm her, as Gaikwad *et al.* (2013) indicated in a study included in the present review that might very well have been the best designed and executed to date. It might also be useful to explain to patients that the anatomical position of the uterus is not the same in lithotomy as in a standing or sitting position (Sunaga *et al.*, 2013). The body of the uterus in a lithotomy position has a more vertical line, which means that embryos might potentially slide down more easily towards the neck, than in a sitting or standing position, in which the body of the uterus is horizontal with the ground, potentially preventing embryos from slipping.

Several aspects suggest that the results of this review are valid. First, we designed a protocol and conducted a comprehensive literature search with no restrictions on design, blinding, sample size, or country of origin. Second, the main outcome and relevant secondary outcomes were clearly defined to explain possible differences between the groups. Finally, potential publication bias was reduced by including data from studies, not publications.

We are fully aware of the several limitations of this study. First, the RCTs with a relatively small number of women included in this meta-analysis might not be sufficient to recognize small differences between groups. There were also differences regarding the inclusion and exclusion criteria between the included studies. The quality of the included trials was moderate due to attrition bias and possible reporting and selection bias. However, it should be mentioned that both LBR and CPR had extremely low heterogeneity, so the final conclusions were not affected. Finally, all studies included data from transfers of more than one embryo; therefore, caution is suggested in extrapolating data to transfers of a single embryo.

## CONCLUSIONS

The findings of this systematic review and meta-analysis suggest that bed rest after ET is not beneficial in terms of achieving a LB. Future randomized controlled studies should focus on the mechanisms through which bed rest might negatively impact ET success rates. In addition, an analysis of the costs related to prolonged bed rest for inpatients or outpatients should be performed, especially in the absence of a direct benefit.

PROSPERO registration number: CRD42020188716
